# Interfacing differently oriented biaxial van der Waals crystals for negative refraction

**DOI:** 10.1515/nanoph-2023-0442

**Published:** 2023-10-20

**Authors:** Ruey-Tarng Liu, Chia-Chien Huang

**Affiliations:** Department of Physics, National Chung Hsing University, Taichung, Taiwan; Department of Physics and Graduate Institute of Nanoscience, National Chung Hsing University, Taichung, Taiwan

**Keywords:** negative refraction, hyperbolic materials, phonon polariton, hybrid plasmon–phonon polariton, image polariton, van der Waals material

## Abstract

Negative refraction has a wide range of applications in diverse fields such as imaging, sensing, and waveguides and typically entails the fabrication of intricate metamaterials endowed with hyperbolic features. In contrast to artificially engineered hyperbolic materials, natural van der Waals (vdW) materials are more accessible owing to their inherent strong in-plane covalent bonding and weak interlayer interactions. However, most vdW materials manifest uniaxial crystal properties, which restrict their behavior solely to out-of-plane hyperbolicity. This characteristic poses a considerable challenge to their seamless integration via planar fabrication techniques, unless a suitable pattern is employed. Recent advances have identified natural biaxial *α*-MoO_3_ as a promising vdW material capable of exhibiting in-plane hyperbolicity. In this study, we performed numerical simulations demonstrating that negative refraction could be achieved by interfacing differently oriented *α*-MoO_3_ slabs coated with tunable graphene on a gold substrate. Our comprehensive analysis yielded three notable outcomes: negative refraction, simultaneous positive and negative refractions, and diffractionless propagation. These outcomes could be operated in a broad range of frequencies and achieved at all angles to offer a superior platform for the flexible manipulation of mid-infrared polaritons. Our findings provide valuable insights into the potential application of other two-dimensional vdW materials for advances in nanoscale super-resolution imaging, molecular sensing, and on-chip photonic integrated circuits.

## 1 Introduction

Negative refraction (NR) is a counterintuitive physical phenomenon where the refracted ray of light is deflected to the same side of the interface normal as the incident ray. NR can be used to realize a superlens capable of achieving a resolution smaller than the wavelength of light, which gives it a wide range of potential applications in super-resolution imaging, molecular sensing, and on-chip nanophotonic waveguides. Over the past two decades, substantial research efforts using diverse structures, materials, and mechanisms have been dedicated to realizing NR in different spectral regimes such as microwaves [1, 2], visible light [3, 4], and near-infrared (IR) light [5–8]. Many studies [1–8] have employed subwavelength dielectrics and metallic elements to artificially construct hyperbolic media [9], which are characterized by permittivity tensors with one component along a principal axis having an opposite sign compared to the other two components. However, fabricating hyperbolic media using bulk materials is a major challenge [1–8].

As an alternative, researchers have turned to natural 2D van der Waals (vdW) materials [10–15], which offer numerous advantages including low-loss characteristics [16, 17], giant optical anisotropy [18, 19], and ultrahigh mode confinement [20–26] for enhanced light–matter interactions. A key advantage of vdW materials is their utilization in creating heterostructures that combine disparate 2D atomic layers without lattice mismatch. These vdW heterostructures [27–32] exhibit hybrid properties derived from the unique advantages of their constituent elements, which provide an additional degree of freedom for manipulating optical properties and designing novel photonic devices. For instance, monolayer graphene and hexagonal boron nitride (hBN) [33] were combined to obtain hybrid plasmon–phonon polaritons (HPPhPs) that combine the advantages of graphene surface plasmon polaritons (GSPPs) in graphene and hyperbolic phonon polaritons (HPhPs) in hBN to demonstrate lower ohmic losses and higher mode confinements than the constituent polaritons. hBN can be used to modify the optical properties of graphene to realize secondary Dirac points or new plasmonic states [34]. Stacking graphene layers with hBN layers overcomes the absence of a bandgap in graphene [35]. Graphene has also been combined with transition metal dichalcogenides (TMDs) [36] to modify the Fermi level and Schottky barrier height, which has resulted in unique properties that are not inherent to either graphene or TMDs.

However, uniaxial hBN and certain TMDs exhibit out-of-plane hyperbolic and in-plane isotropic dispersions. This behavior arises from the permittivity tensors, which possess two negative components in the in-plane direction and a positive component in the out-of-plane direction [37, 38]. The out-of-plane hyperbolicity poses a major challenge for the design of planar photonic circuits. hBN and TMDs require nanostructured patterning to achieve in-plane hyperbolicity [39–41], but this introduces surface roughness and defects that increase optical losses. Specifically, the reconfigurable topological features of hyperbolic polariton vortices [42] induced in pristine hBN flakes are also reported to tailor spin–orbit interactions.

Recently, a natural biaxial vdW semiconductor has been discovered that exhibits in-plane hyperbolic dispersion: *α*-phase molybdenum trioxide (*α*-MoO_3_) [43–46]. A twisted bilayer [47–51] and interface engineering [52, 53] of the structure have been proposed to further manipulate the properties of *α*-MoO_3_ and realize exotic phenomena such as topological transitions, wavefront control, directional canalization, and diffractionless propagation. By covering the *α*-MoO_3_ slabs with monolayer graphene, their optical properties can be tuned by varying the charge carrier density of the graphene [54–56]. Two independent groups recently demonstrated NR at the interface of vdW heterostructures. Hu et al. [57] utilized *α*-MoO_3_ slabs partially covered with graphene while Sternbach et al. [58] stacked *α*-MoO_3_ slabs with type-II hyperbolicity and h^11^BN slabs with type-I hyperbolicity. In this study, we propose a novel mechanism of interfacing differently oriented *α*-MoO_3_ slabs covered with a tunable graphene layer on an Au substrate to achieve broadband and all-angle NR. Notably, the use of an Au substrate provides significantly enhanced mode confinement of the polaritons compared to a SiO_2_ substrate. Our proposed mechanism presents an innovative platform for effectively manipulating mid-IR polaritons for further advances in the development of polaritonic devices.

## 2 Methods

### 2.1 Proposed structure

**Figure 1:**
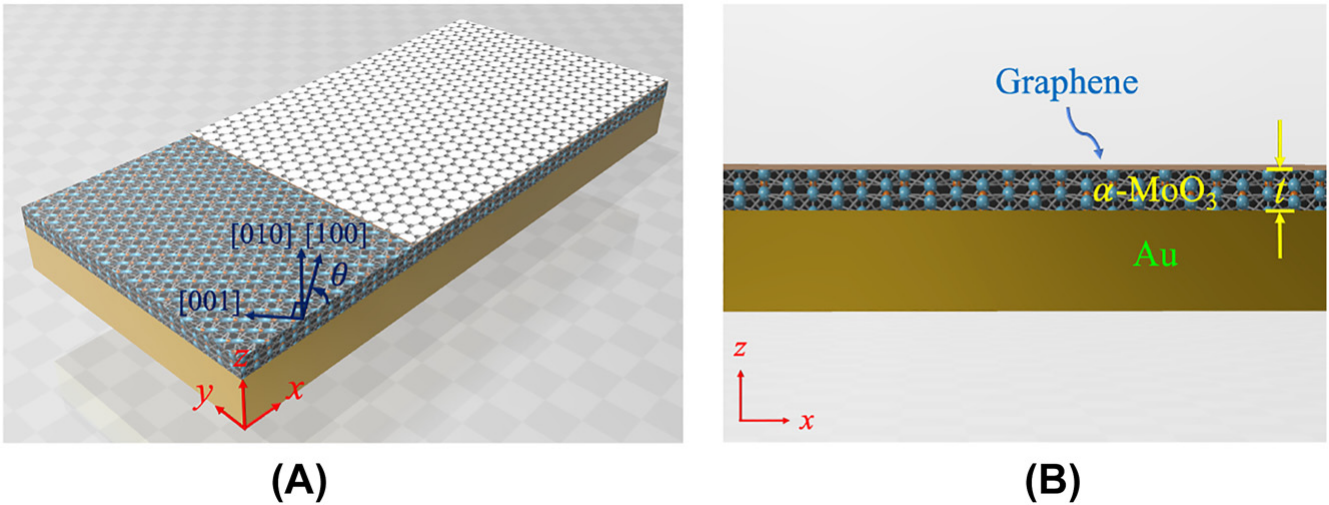
The structure with single *α*-MoO_3 _slab. (A) Illustration of the air/graphene/*α*-MoO_3_/Au structure where [100], [001], and [010] are the principal axes of the biaxial *α*-MoO_3_ slab and *θ* is the angle between the *x*-axis and [100] direction. (B) The *x*–*z* plane of the structure, denoted as (A), where *t* is the thickness of the *α*-MoO_3_ slab.

Figure 1A and B illustrate the proposed structure and its cross-sectional view (*x*–*z* plane), respectively, which comprises air/graphene/*α*-MoO_3_ on an Au substrate. Here, *t* is the thickness of the *α*-MoO_3_ slab, [100] and [001] are the principal axes of the *α*-MoO_3_ crystal, and *θ* is the angle between the *x*-axis and [100] direction. The employment of a metal substrate helps the proposed structure realize significantly higher mode confinement than the conventional HPhP, which is referred to as a hyperbolic image plasmon–phonon polariton (HIPPhP) [23]. The HIPPhP originates from the hybridization of a hyperbolic polariton in the *α*-MoO_3_ slab and their mirror image with an inverted charge distribution in the perfect electric conductor (PEC) [21], which behaves as a mirror. At the mid-IR band, Au can be considered an approximate PEC. Therefore, most of the electric field is confined in the *α*-MoO_3_ slab, which significantly enhances the mode confinement due to the opposing charge distributions of the original and mirror polaritons.

### 2.2 Numerical simulations

We specifically investigated the reststrahlen band II (RB II) of *α*-MoO_3_, which encompasses the frequency range of 816–972 cm^−1^. Within this frequency range, the permittivity (*ε*) components along the principal axes [100], [001], and [010] exhibit the characteristics of *ε*_*x*_ < 0, *ε*_*y*_ > 0, and *ε*_*z*_ > 0, respectively. The RB is a range of wavelengths where a material exhibits strong absorption due to the resonance between the incident radiation and lattice vibration. Furthermore, an HPhP occurs when the incident radiation couples strongly with the oscillations of the lattice vibrations in the *α*-MoO_3_ slab. Therefore, the RB can give rise to hyperbolic dispersion and the formation of hyperbolic polaritons in the *α*-MoO_3_ slab. Notably, the negative sign of Re (*ε*_*x*_) × Re (*ε*_*y*_) < 0 implies that the *α*-MoO_3_ has in-plane hyperbolicity in the *x*–*y* plane. The complex permittivity of Au can be obtained from the literature [59]. Here, the graphene sheet was modeled as an infinitely thin layer with a surface current density. The surface conductivity of graphene can be calculated by using the Kubo formula [60] (see Supplementary Material, §1). A numerical model was constructed in COMSOL Multiphysics with the mesh resolution set sufficiently fine and the computational domain sufficiently large to ensure simulation accuracy. The permittivity of *α*-MoO_3_ was obtained by employing the conventional Lorentz model [38, 43, 44, 58] (see Supplementary Material, §2.1). The crystallographic directions [100], [001], and [010] of *α*-MoO_3_ coincided with the coordinates *x*, *y*, and *z*, respectively, under the condition *θ* = 0°. The real and imaginary components of the permittivity of *α*-MoO_3_ were plotted (see Supplementary Material, Figure S1). We placed an electric dipole source with polarization along the *z*-direction at a position 50 nm above the top graphene layer. The electric fields, represented as Re(*E*_*z*_), were calculated at a probing height of 20 nm above the graphene layer. To obtain the corresponding IFCs in wavevector space (*k*_*x*_, *k*_*y*_), we performed a Fourier transform of the Re(*E*_*z*_) fields using spatial Fast Fourier Transform (FFT) with spatial sampling resolutions set to *N*_*x*_ = 1000 and *N*_*y*_ = 1000. To minimize undesired reflections, the computational window was enclosed by a perfect-matched-layer boundary condition with a thickness of 1 µm, effectively absorbing the transmitted waves. Simulations were performed to analyze the effects of modifying the *α*-MoO_3_/Au structure with graphene, the effects of interfacing α-MoO_3_ slabs at different orientations, and the effects of multiple interfaces.

## 3 Results

### 3.1 Polariton modes with and without graphene

To gain insights into the mode characteristics, the dispersion properties of the *α*-MoO_3_/Au structure at *t* = 150 nm were analyzed. The principal crystallographic direction [100] was aligned with the *x*-direction (i.e., *θ* = 0°). The results indicated an electromagnetic field with unprecedented confinement (see Supplementary Material, Figure S2A). The relationship between the propagation length (*L*_*p*_) and *t* (see Figure S2B) was found to follow the conventional tradeoff between mode confinement and propagation loss, where an increase in *t* leads to looser mode confinement and longer *L*_*p*_. The figure of merit [FOM = Re(*k*_*x*_)/Im(*k*_*x*_)] was obtained to assess the benefit–cost ratio between mode confinement and propagation loss [22, 46]. High FOM values were obtained at the wavenumbers *ω* = 880–920 cm^−1^ (see Figure S2C), which indicates a favorable tradeoff in this frequency range. The group velocity (*V*_*g*_) (see Figure S2D) exhibited a similar trend to *L*_*p*_. The field distributions of the HIPPhP (see Supplementary Material, Figure S3) clearly demonstrated tighter confinement and a shorter wavelength as the thickness decreased.

**Figure 2:**
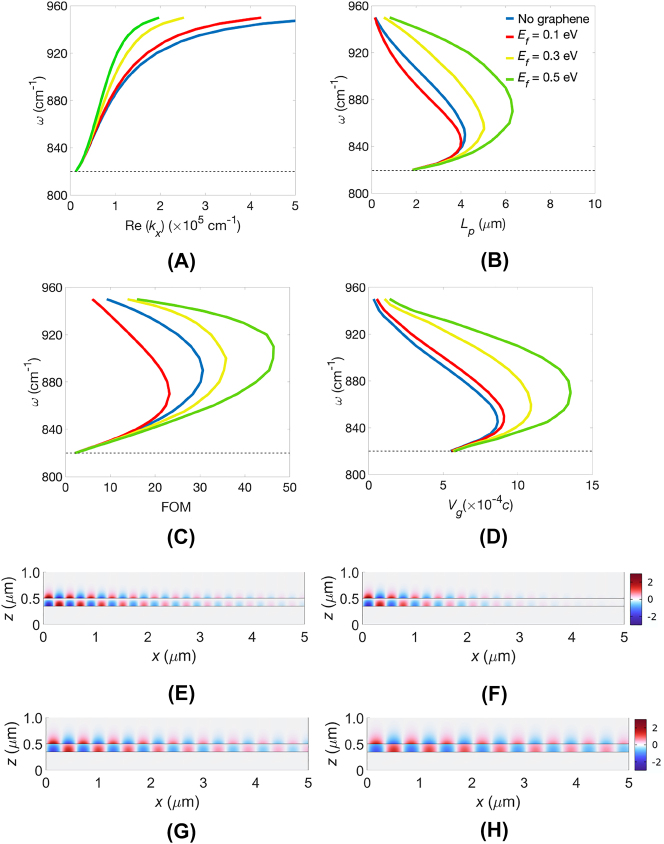
Wavenumber *ω* of an *α*-MoO_3_ slab at *t* = 150 nm and covered with monolayer graphene at several values of *E*_*f*_. against (A) Re(*k*_*x*_), (B) *L*_*p*_, (C) FOM, and (D) *V*_*g*_. *E*_*z*_ field profiles of an *α*-MoO_3_ slab with *t* = 150 nm: (E) no graphene and covered by monolayer graphene at (F) *E*_*f*_ = 0.1 eV, (G) *E*_*f*_ = 0.3 eV, and (H) *E*_*f*_ = 0.5 eV.

Next, the dispersion properties of the graphene/*α*-MoO_3_/Au structure were obtained. Increasing the Fermi energy (*E*_*f*_) was found to decrease Re(*k*_*x*_) with a more pronounced dependence at higher frequencies (see Figure 2A). In contrast, *L*_*p*_, FOM, and *V*_*g*_ all increased (see Figure 2B–D). A higher FOM indicates a stronger mode confinement or lower mode loss. Interestingly, when *E*_*f*_ is less than 0.1 eV, the case without graphene exhibits the strongest mode confinement but also the largest energy loss. Conversely, when *E*_*f*_ exceeds 0.2 eV, the situation reverses, resulting in a smaller FOM (see Figure 2C). This phenomenon can be attributed to plasmons reaching the interband transition threshold at very low *E*_*f*_ for specific wavevectors, leading to overdamped outcomes [61, 62]. Notably, the value of *E*_*f*_ reaching the interband transition threshold decreases as the wavevector reduces. In contrast, plasmons transition to the intraband transition as *E*_*f*_ increases. As a result, the HIPPhP modes have larger FOM when *E*_*f*_ > 0.2 eV and smaller when *E*_*f*_ < 0.1 eV while comparing to the case without graphene for the condition given in the proposed system. The Re(*E*_*z*_) distributions at different values of *E*_*f*_ (see Figure 2E–H) are also shown that both polariton wavelength and *L*_*p*_ increased with increasing *E*_*f*_. These results offer valuable insights into the influence of graphene doping and its effects on the field propagation characteristics of the HIPPhP.

### 3.2 Effect of different orientations

We first analyzed a single *α*-MoO_3_ slab rotated counterclockwise around the *z*-axis by the angle *θ*, as shown in Figure 1A. Here, *θ* corresponds to the orientation of the crystallographic direction [100] with respect to the *x*-axis. We used this coordinate system to obtain the anisotropic permittivity tensor of the *α*-MoO_3_ slab (see Supplementary Material, §2.1) under the conditions of *ω* = 910 cm^−1^, *t* = 150 nm, and *E*_*f*_ = 0.15 eV. The Re(*E*_*z*_) field distributions varied with *θ* and demonstrated directional canalization along the corresponding rotation angles (see Figure S4). These results provide compelling evidence of the in-plane hyperbolicity of the HIPPhP. To analyze the wavevector properties of the Re(*E*_*z*_) fields, we performed a Fourier transform and plotted the resulting isofrequency contours (IFCs) in wavevector space (*k*_*x*_, *k*_*y*_) (see Figure S5). The amplitude of the IFCs reflected the energy distribution in the corresponding wavevector space. Notably, a stronger amplitude indicates a higher concentration of energy. At *θ* = 0°, a significant portion of the energy was concentrated around *k*_*y*_/*k*_0_ = −100 to 100, while *k*_*x*_/*k*_0_ = ±20. The symmetric axis of the IFCs (i.e., *k*_*x*_/*k*_0_ = 0) exhibited a tilt as *θ* was varied, which implies that the directional propagation of energy can be flexibly tuned by adjusting *θ*. To validate the accuracy of the numerically calculated IFCs, we superimposed them with analytically calculated IFCs obtained by solving a four-layer waveguide system (air/graphene/MoO3/Au), as described in reference [58], for the first three cases (refer to Figure S5A–C). The inner elliptical and outer hyperbolic IFCs, indicated by green dashed lines, represent the fundamental and first-order HIPPhP modes. It is evident that there is excellent agreement between the results obtained from the numerical and analytical IFCs. To explore the tunability of the system, we obtained the Re(*E*_*z*_) field and corresponding IFC for a bare *α*-MoO_3_ slab and graphene/*α*-MoO_3_ slab with different values of *E*_*f*_ at *θ* = 20° (see Figure S6). We observed canalization of the field distributions at different doping levels. Notably, the opening angle of the hyperbola asymptote of the IFC increased with *E*_*f*_, and the bare *α*-MoO_3_ slab exhibited the smallest opening angle. This behavior aligns consistently with the field distribution patterns. The Re(*E*_*z*_) field profiles showed the emergence of a hyperboloid-shaped profile along the [100] direction and ellipse-shaped profile along the [001] direction. As *E*_*f*_ was increased to 0.3 eV, the elliptical distribution became dominant. The variations in the field profiles were also reflected in the IFCs, which indicates a doping-driven topological transition from the manipulation of *E*_*f*_ [58]. Notably, increasing *E*_*f*_ reduced the size of the elliptical profile in the IFCs.

**Figure 3:**
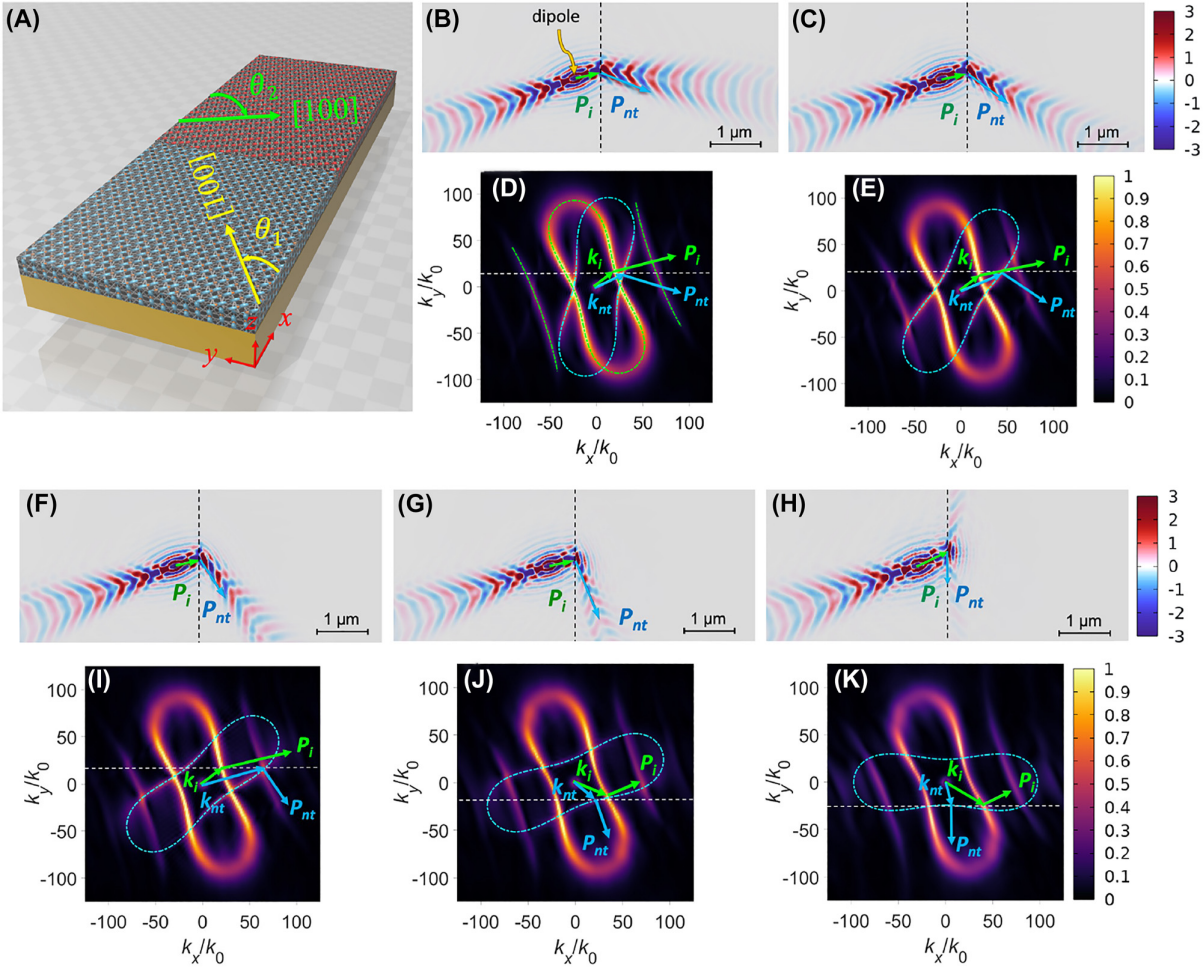
The structure with two differently oriented *α*-MoO_3_ slabs. (A) Graphene-covered *α*-MoO_3_ slabs with different crystal orientations on an Au substrate, where *θ*_1_ and *θ*_2_ are the angles between the *x*-axis and [100] directions of the individual slabs. The left and right *α*-MoO_3_ slabs are indicated by light green and light red colors, respectively. Re(*E*_*z*_) field distributions under the conditions of *ω* = 910 cm^−1^, *t* = 150 nm, *E*_*f*_ = 0.15 eV, and *θ*_1_ = 20° at *θ*_2_ of (B) −10°, (C) −30°, (F) −50°, (G) −70°, and (H) −90° and (D), (E), (I)–(k) the corresponding IFCs, where the green and cyan dashed lines indicate the analytical calculated IFCs of the left and right *α*-MoO_3_ slabs, respectively.

Next, we investigated NR when two *α*-MoO_3_ slabs, both covered by monolayer graphene, were interfaced with different orientations. Figure 3A depicts the left and right slabs in light green and light red, respectively. The rotation angles between the *x*-axis and [100] directions of the individual slabs were denoted as *θ*_1_ and *θ*_2_. Under the conditions of *ω* = 910 cm^−1^, *t* = 150 nm, *E*_*f*_ = 0.15 eV, and *θ*_1_ = 20°, we investigated the Re(*E*_*z*_) field distributions and corresponding IFCs for *θ*_2_ of −10° to −90°. Figure 3B, C, and F–H depict the field distributions of Re(*E*_*z*_) with *P*_*i*_ and *P*_*nt*_. The black dashed line indicates the interface between the two slabs. No significant power scattering or reflection occurred at the interfaces within the range of *θ*_2_ = −10° to −90°. Figure 3D, E, and I–K depict the corresponding IFCs where they overlapped. Here, we have superimposed the analytically calculated IFCs onto the numerical results, as illustrated in Figure 3D, E, and I–K. In the case of the left *α*-MoO_3_ slab with a fixed orientation angle of *θ*_1_ = 20°, we have indicated the inner elliptical and outer hyperbolic IFCs using green dashed lines. For the right *α*-MoO_3_ slab, we have indicated the elliptical IFCs with different angles of *θ*_2_ using cyan dashed lines. Note that the hyperbolic IFCs associated with the first-order HIPPhP modes of the right *α*-MoO_3_ slab are not visible due to their significantly weaker amplitudes. The calculated IFCs for the right slab exhibited inclined orientations that varied with *θ*_2_. The trajectories of the incident (*k*_*i*_) and refracted (*k*_*nt*_) wavevectors are depicted with dashed green and red lines, respectively, while the incident (*P*_*i*_) and refracted (*P*_*nt*_) Poynting vectors (normal to the IFCs) are represented by solid green and red lines, respectively. The dashed white line represents the fixed *y*-component (*k*_*y*_) of the incident wavevector, which was preserved as light passed through the interface. Notably, *P*_*nt*_ was refracted toward the opposite side of the interface normal to *P*_*i*_. Consequently, NR was achieved by manipulating the crystal orientations of the *α*-MoO_3_ slab, and the NR angle can be arbitrarily determined by *θ*_2_. These results demonstrate the ability of the proposed structure to achieve all-angle NR in the mid-IR regime.

Furthermore, we examined the dependence of NR on the thickness of the *α*-MoO_3_ slab. Figure S7 shows the Re(*E*_*z*_) fields at different values of *t* and corresponding IFCs under the conditions of *ω* = 910 cm^−1^, *E*_*f*_ = 0.15 eV, *θ*_1_ = 20°, and *θ*_2_ = −30°. Please note that the outer hyperbolic IFCs of the right *α*-MoO_3_ slab with a thickness of 200 nm (see Figure S7K) are also represented by cyan dashed lines that is the same color used for its inner elliptical IFCs. The NR angle increased moderately with increasing *t*. This behavior can be explained by considering the formation of the resultant HIPPhP, which arises from the coupling of a GSPP with an elliptical IFC and HIPhP with a hyperbolic IFC. The contribution of the GSPP to the resultant mode of the *α*-MoO_3_ slab increases with decreasing *t*. However, decreasing *t* also lowers the threshold *E*_*f*_ required for the topological transition to occur [56], which weakens the hyperbolicity of the resultant HIPPhP at a fixed *E*_*f*_. Thus, the largest NR angle was observed at *t* = 300 nm, as shown in Figure S7L. In addition to *E*_*f*_, *t* provides an additional parameter for controlling the occurrence of the topological transition. Therefore, while mode canalization for any given thickness of *α*-MoO_3_ can be adjusted through graphene doping, this adjustment is accompanied by a notable reduction in field strength along the canalization direction, while simultaneously resulting in a stronger field propagating in all directions. To study the dependence of NR on frequency, the Re(*E*_*z*_) fields for different operating frequencies within the RB II of *α*-MoO_3_ along are shown in Figure S8A–F with the corresponding IFCs (see Figure S8G–L). Increasing *ω* from 830 to 930 cm^−1^ cause the IFC patterns to transform gradually from a closed-form ellipse to an open-form hyperbola. The broadband and the all-angle NRs highlight the potential of this system for realizing NR over a broad range of frequencies and incident angles.

### 3.3 Effect of multiple interfaces

**Figure 4:**
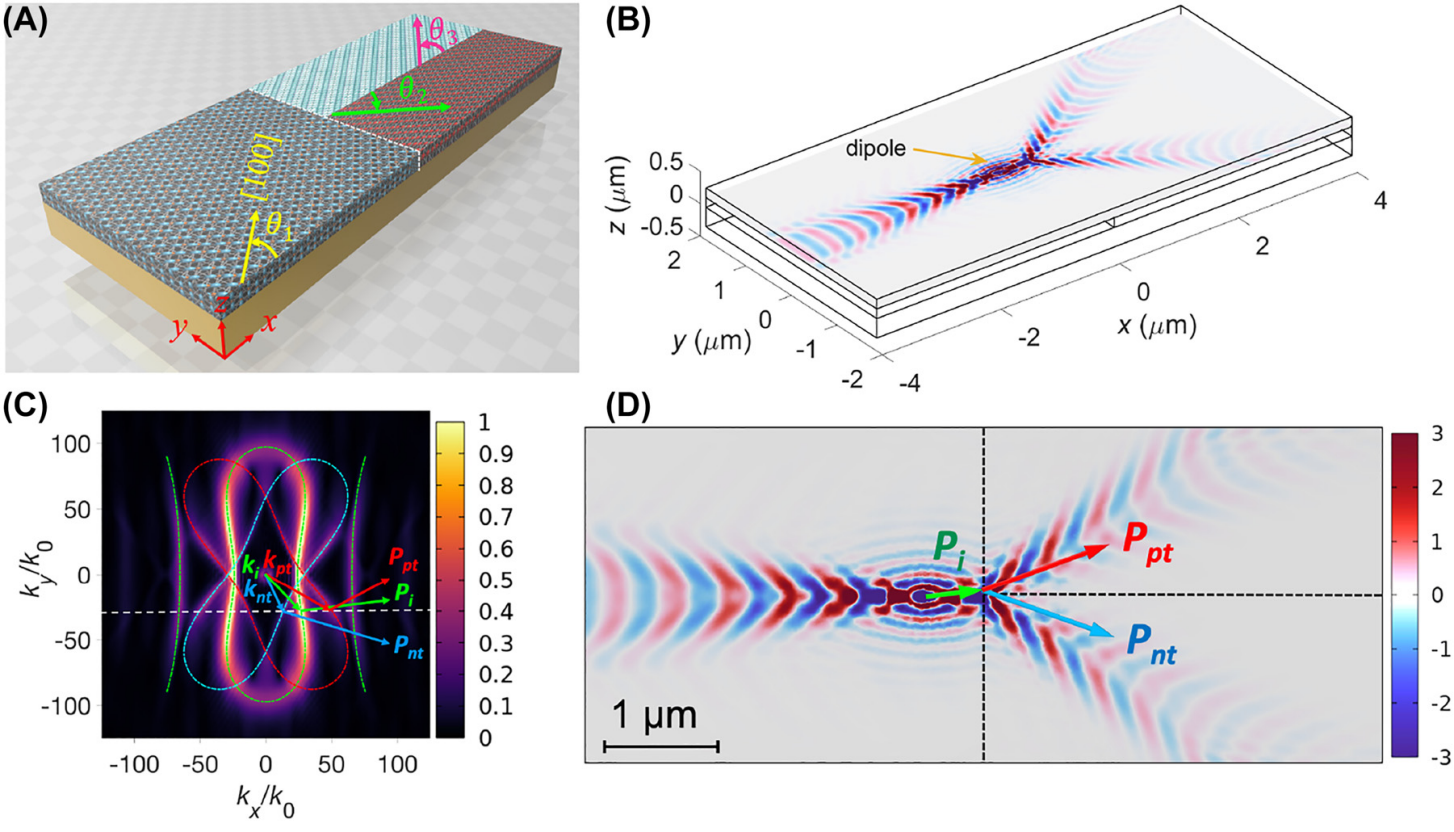
The structure with three differently oriented *α*-MoO_3_ slabs. (A) Interfacing three *α*-MoO_3_ slabs with crystal orientations *θ*_1_, *θ*_2_, and *θ*_3_ between the *x*-axis and [100] directions of the individual slabs. (B) 3D view of the Re(*E*_*z*_) field distribution. (C) IFC with the incident wavevector (*k*_*i*_), two transmitted wavevectors (*k*_*pt*_ and *k*_*nt*_), and the incident (*P*_*i*_), positive refractive (*P*_*pt*_), and negative refractive (*P*_*nt*_) poynting vectors, where the green, red, and cyan dashed lines indicate the analytical calculated IFCs of the left, upper-right, and lower-right *α*-MoO_3_ slabs, respectively. (D) Top view of the Re(*E*_*z*_) field distribution.

Figure 4A shows the extension of the proposed structure to multiple interfaces comprising three *α*-MoO_3_ slabs with different crystal orientations. The angles between the *x*-axis and [100] directions of the left, upper-right, and lower-right slabs are denoted as *θ*_1_, *θ*_2_, and *θ*_3_, respectively. This configuration allowed the crystal orientations of each *α*-MoO_3_ slab to be manipulated and introduced additional degrees of freedom for controlling the optical properties of the system. The angles *θ*_1_, *θ*_2_, and *θ*_3_ can be tuned to engineer the dispersion, wave propagation, and NR characteristics to realize various intriguing optical phenomena and functionalities. Figure 4B shows a three-dimensional view of the Re(*E*_*z*_) field distribution under the conditions *ω* = 910 cm^−1^, *t* = 150 nm, *E*_*f*_ = 0.15 eV, *θ*_1_ = 0°, *θ*_2_ = −30°, and *θ*_3_ = 30°. The in-plane coordinate of a *z*-polarized electric dipole was located at (−0.5, 0), where the crossing point of the two interfaces served as the origin. As the polariton passed through the origin, it was split and refracted into two preset transmission directions corresponding to *θ*_2_ = −30° and *θ*_3_ = 30°. Figure 4C visualizes *k*_*i*_ and the two transmitted wavevectors *k*_*pt*_ and *k*_*nt*_, where *k*_*pt*_ is the positive refraction in the IFCs derived from the three differently oriented *α*-MoO_3_ slabs. The analytical IFCs representing the fundamental modes of the left, upper-right, and lower-right α-MoO3 slabs are denoted by green, red, and cyan, respectively. Additionally, only the hyperbolic IFCs associated with the first-order mode of the left *α*-MoO3 slab are observable. The corresponding *P*_*i*_, *P*_*pt*_, and *P*_*nt*_ are also indicated, where *P*_*pt*_ is the positive refractive Poynting vector. The three overlapping IFCs resulted from the three interfaces between the *α*-MoO_3_ slabs. Figure 4D shows a top view of the Re(*E*_*z*_) field distribution and highlights the power flows represented by the Poynting vectors *P*_*i*_, *P*_*pt*_, and *P*_*nt*_. In this structure, positive refraction and NR coexist, which allows it to function as a nanoscale beam splitter in photonic integrated circuits. The proposed design can serve as a functional Mach–Zehnder modulator [63] while interfacing seven *α*-MoO_3_ slabs with various orientations. This demonstrates the versatility and applicability of the proposed structure for different types of photonic devices.

**Figure 5:**
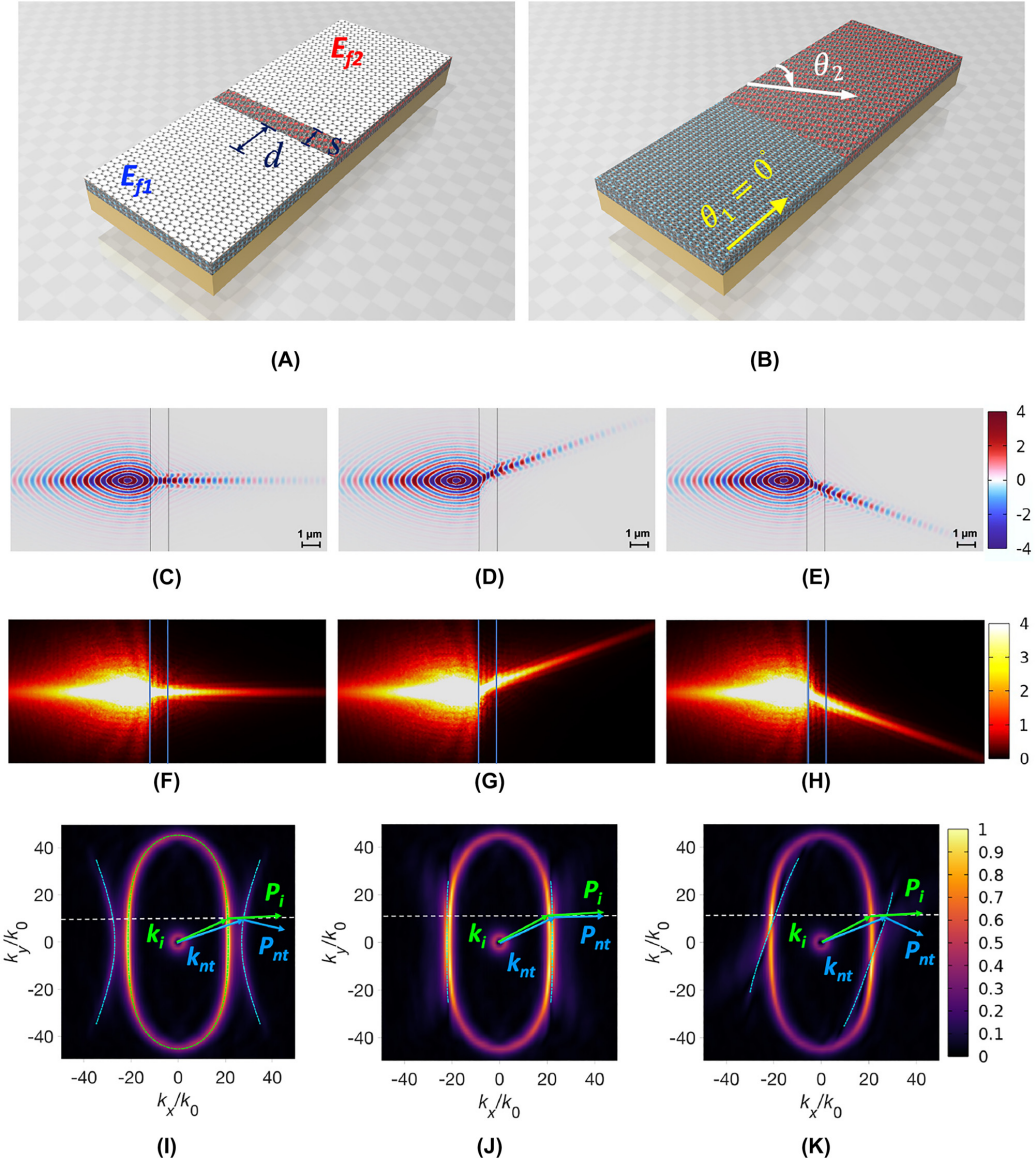
The structure with two differently oriented *α*-MoO_3_ slabs partially covered with graphene sheet. (A) Interfacing two *α*-MoO_3_ slabs, in which the left slab is entirely covered with graphene (*E*_*f*1_) but the right slab is partially covered with graphene (*E*_*f*2_). (B) The same as (A), except peeling off the graphene layer to clearly show the orientations of *α*-MoO_3_ slabs. The two graphene layers have a separation distance of *S* = 1 µm, where *θ*_1_ = 0° (left slab) and *θ*_2_ = 0° or −20° (right slab) indicate the angles between the *x*-axis and [100] directions of the individual slabs. Re(*E*_*z*_) field distributions for various cases: (C) *θ*_1_ = 0° and *θ*_2_ = 0° [56] without the graphene layer (*E*_*f*2_) covering the right *α*-MoO_3_ slab, (D) *θ*_1_ = 0° and *θ*_2_ = 0°, and (E) *θ*_1_ = 0° and *θ*_2_ = −20°. (F)–(H) Corresponding |**E**| fields. (I)–(K) Corresponding IFCs, where the green and cyan dashed lines indicate the analytical calculated IFCs of the left and right *α*-MoO_3_ slabs, respectively.

To further control the polaritons, we interfaced two *α*-MoO_3_ slabs partially covered with graphene, as shown in Figure 5A. The left *α*-MoO_3_ slab had a crystal orientation of *θ*_1_ = 0°, as shown in Figure 5B, and was completely covered with monolayer graphene, which was electrically gated with *E*_*f*1_. The right slab had the crystal orientation *θ*_2_, and it was partially covered with graphene, which was electrically gated with *E*_*f*2_. The separation between the graphene layers was denoted as *S*. A *z*-polarized electric dipole was positioned at a distance of *d* = 1 µm from the left interface on the *x*–*y* plane. The other conditions were set to *ω* = 910 cm^−1^, *t* = 150 nm, *S* = 1 µm, *E*_*f*1_ = 0.3 eV, *θ*_1_ = 0°, and *E*_*f*2_ = 0.25 eV. Figure 5D and E display the Re(*E*_*z*_) field distributions for *θ*_2_ = 0° and −20°, respectively. As a comparison, Figure 5C illustrates the results when only the left *α*-MoO_3_ slab was covered with graphene with both *θ*_1_ and *θ*_2_ set to 0° [58]. The |**E**| fields and IFCs for the three cases are also shown. Figure 5C corresponds to the structure proposed by Hu et al. [58], which exhibited NR (Figure 5I) and planar focusing (Figure 5F). However, after passing through the focal point, the field experienced conventional diffraction. In contrast, partially covering the graphene on the right *α*-MoO_3_ slab with a suitable *E*_*f*2_ = 0.25 eV overcame the diffraction of the light beam, and diffractionless propagation was achieved (Figure 5D and G). In addition, the limitation of a fixed focal point [58] was eliminated. This achievement opens up the possibility for diffractionless propagation in imaging systems and steering the diffractionless beam in wide angles to any desired direction by presetting the crystal orientation (Figure 5E and H). The IFCs for Figure 5D and E are also shown in Figure 5J and K, respectively. The parameters *d*, *S*, *E*_*f*1_, and *E*_*f*2_ can be adjusted to realize different imaging capabilities.

## 4 Conclusions

In this study, we demonstrated the feasibility of interfacing differently oriented *α*-MoO_3_ slabs covered with tunable graphene on an Au substrate to realize NR. We provide compelling evidence for the presence of NR across various structures. By tailoring the orientations of the *α*-MoO_3_ slabs and modulating the Fermi energy of the graphene layer, three remarkable outcomes were achieved: broadband and all-angle NR, simultaneous positive refraction and NR, and diffractionless propagation for flexible manipulation of mid-infrared polaritons. The integration of multiple *α*-MoO_3_ slabs opens up exciting approaches for designing and implementing photonic devices such as beam splitters and Mach–Zehnder modulators. The significance of these findings extends beyond the materials and structures considered in this study as they are potentially applicable to other types of vdW materials.

## Supplementary Material

Supplementary Material Details

## Abbreviations

FOM: figure of merit
FT: Fourier transform
GSPP: graphene surface plasmon polaritons
hBN: hexagonal boron nitride
HPhP: hyperbolic phonon polariton
HPPhP: hybrid plasmon–phonon polariton
IFC: isofrequency contour
NR: negative refraction
PEC: perfect electric conductor
RB: reststrahlen band
TMD: transition metal dichalcogenides
vdW: van der Waals
