# Supplementary material for: Interfacing differently oriented biaxial van der Waals crystals for negative refraction
Source: Nanophotonics. 2023 Oct 20;12(21):4063–72. doi: 10.1515/nanoph-2023-0442 (PMC11501674; doi:10.1515/nanoph-2023-0442)
Supplement: Supplementary file 1 — Supplementary Material Details [file j_nanoph-2023-0442_suppl_001.docx]

Supplementary Material

Ruey-Tarng Liu and Chia-Chien Huang[[1]](#footnote-1)*

Interfacing Differently Oriented Biaxial van der Waals Crystals for Negative Refraction

1. Surface conductivity of graphene

The surface conductivity of graphene *σ* was calculated by using the Kubo formula [60]:

(S1)

where *ω* is the angular frequency, *E_f_* is the Fermi energy, *τ* = *µE_f_* /*ev_f_* ^2^ is the carrier relaxation lifetime, *T* is the absolute temperature, *e* is the electron charge, *k_B_* is the Boltzmann constant, *ℏ* is the reduced Plank constant, *µ* is the carrier mobility in graphene, and *v_f_* = 10^6^ m/s is the Fermi velocity of electrons. The optical properties of graphene can be tuned by adjusting *E_f_* via electrical gating or chemical doping. The carrier mobility of graphene ranges from *µ*> 0.1 m^2^/V·s with typical chemical vapor deposition to *µ*> 20 m^2^/V·s with suspended exfoliation. For our calculations, we adopted a practical value of *µ* = 1 m^2^/V·s at *T* = 300 K.

1. Permittivity of the *α*-MoO_3_ slab
   1. No rotation

The permittivity of *α*-MoO_3_ was calculated by employing the following Lorentz model [38,43,44,58]:

$\varepsilon_{k}(\omega)=\varepsilon_{\infty}^{k}\left(1+\frac{\omega_{LO}^{k\text{ 2}}-\omega_{TO}^{k\text{ 2}}}{\omega_{TO}^{k\text{ 2}}-\omega^{2}-i\omega\gamma^{k}}\right),\text{ }k=x,y,z$ (S2)

where 𝜀*_k_* is the principal component of the permittivity tensor of *α*-MoO_3_; $\varepsilon_{\infty}^{k}$ ( ) corresponds to the high-frequency dielectric constants along the *k* direction; $\omega_{LO}^{k}$ ( ) and$\omega_{TO}^{k}$ ( ) refer to the LO and TO phonon frequencies, respectively; and $\gamma^{k}$($\gamma^{x}=4,$ $\gamma^{y}=4,$ $\gamma^{z}=2$) is the inelastic loss rate of the material. **Figure S1** plots the real (Re(*ε*)) and imaginary (Im(*ε*)) components of the permittivity of *α*-MoO_3_.


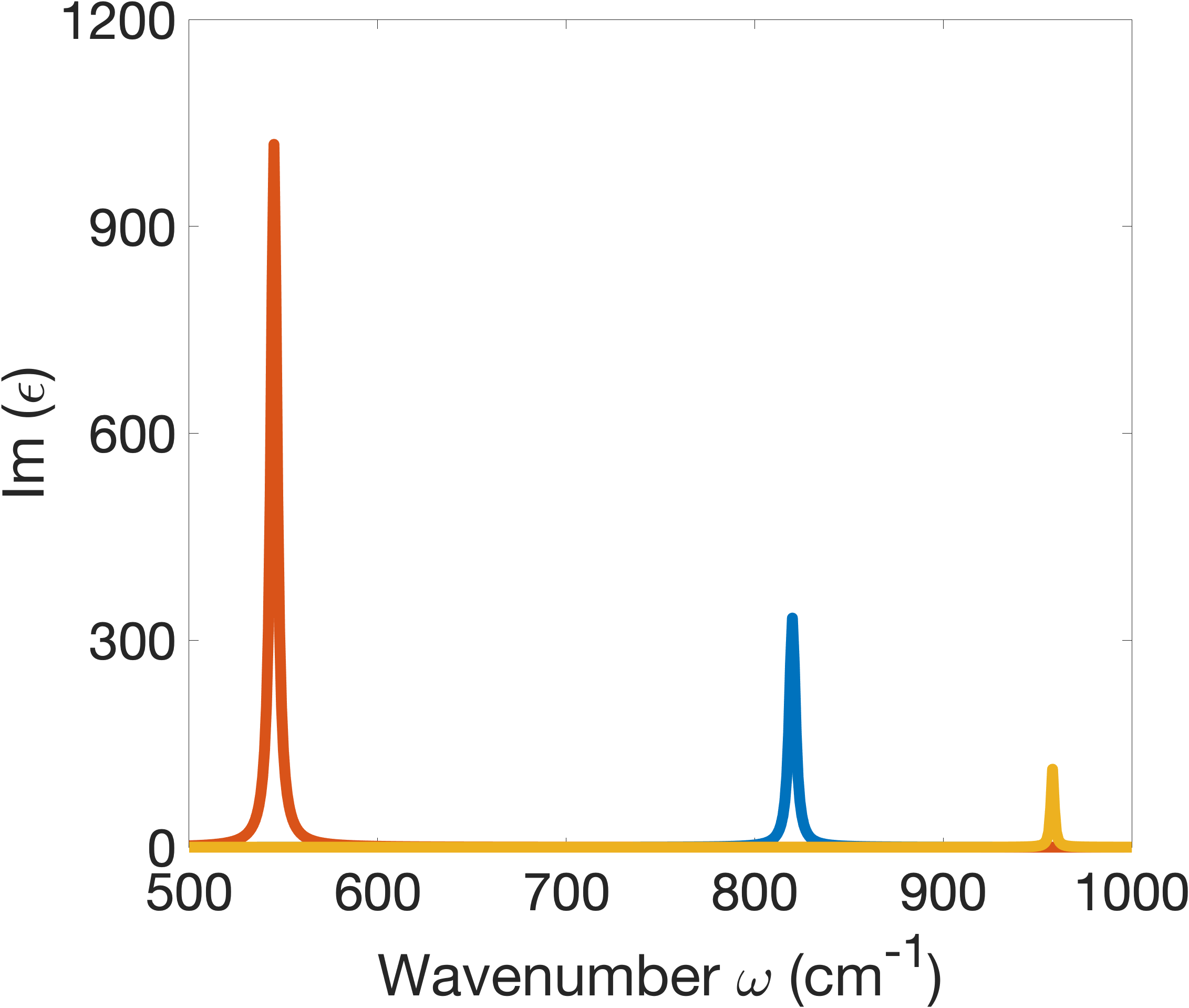


(A) (B)

**Fig. S1:** (A) Real and (B) imaginary components of the permittivity tensor (*ε*) of *α*-MoO_3_ along the three principal axes of [100], [001], and [010]; RB II is drawn in a translucent green color.

- 1. Rotated by the angle *θ*

By rotating an *α*-MoO_3_ slab counterclockwise around the *z*-axis by the angle *θ* (i.e., the orientation of the principal axis [100] with respect to the *x*-axis), the anisotropic permittivity tensor of the slab can be expressed as follows:

$\left[\begin{matrix} \varepsilon_{x}\cos^{2}\theta+\varepsilon_{y}\sin^{2}\theta& \left(\varepsilon_{y}-\varepsilon_{x}\right)\cos\theta\sin\theta& 0 \\ \left(\varepsilon_{y}-\varepsilon_{x}\right)\cos\theta\sin\theta& \varepsilon_{y}\cos^{2}\theta+\varepsilon_{x}\sin^{2}\theta& 0 \\ 0 & 0 & \varepsilon_{z} \end{matrix}\right],$ (S3)

where *ε_x_*, *ε_y_*, and *ε_z_* are the permittivity components along the principal axes [100], [001], and [010], respectively, of the *α*-MoO_3_ slab and *θ* is positive for counterclockwise rotation from the *x*-axis.

- 1. Dispersion properties and Re(*E_z_*) field distributions of *α*-MoO_3_/Au structure


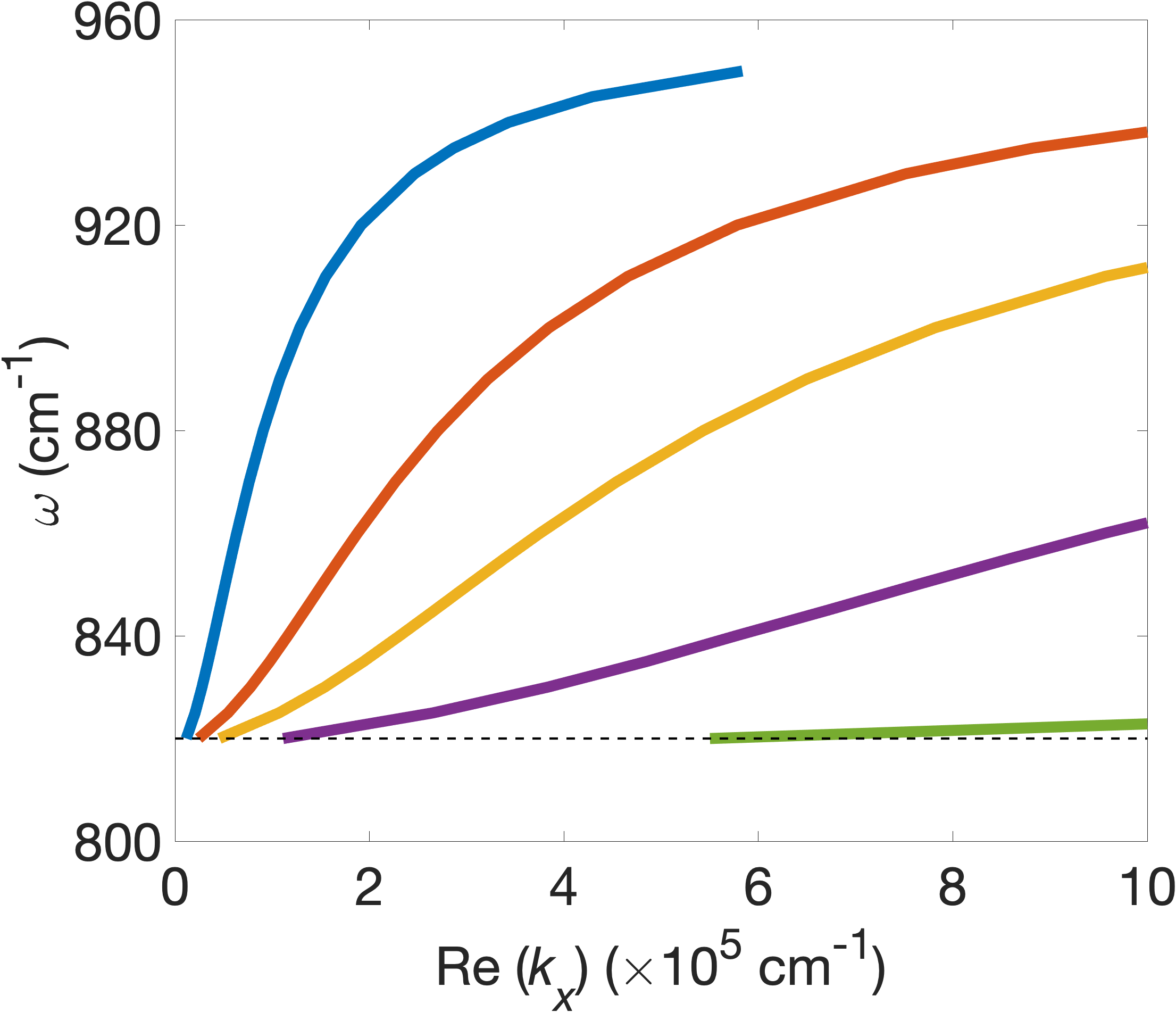

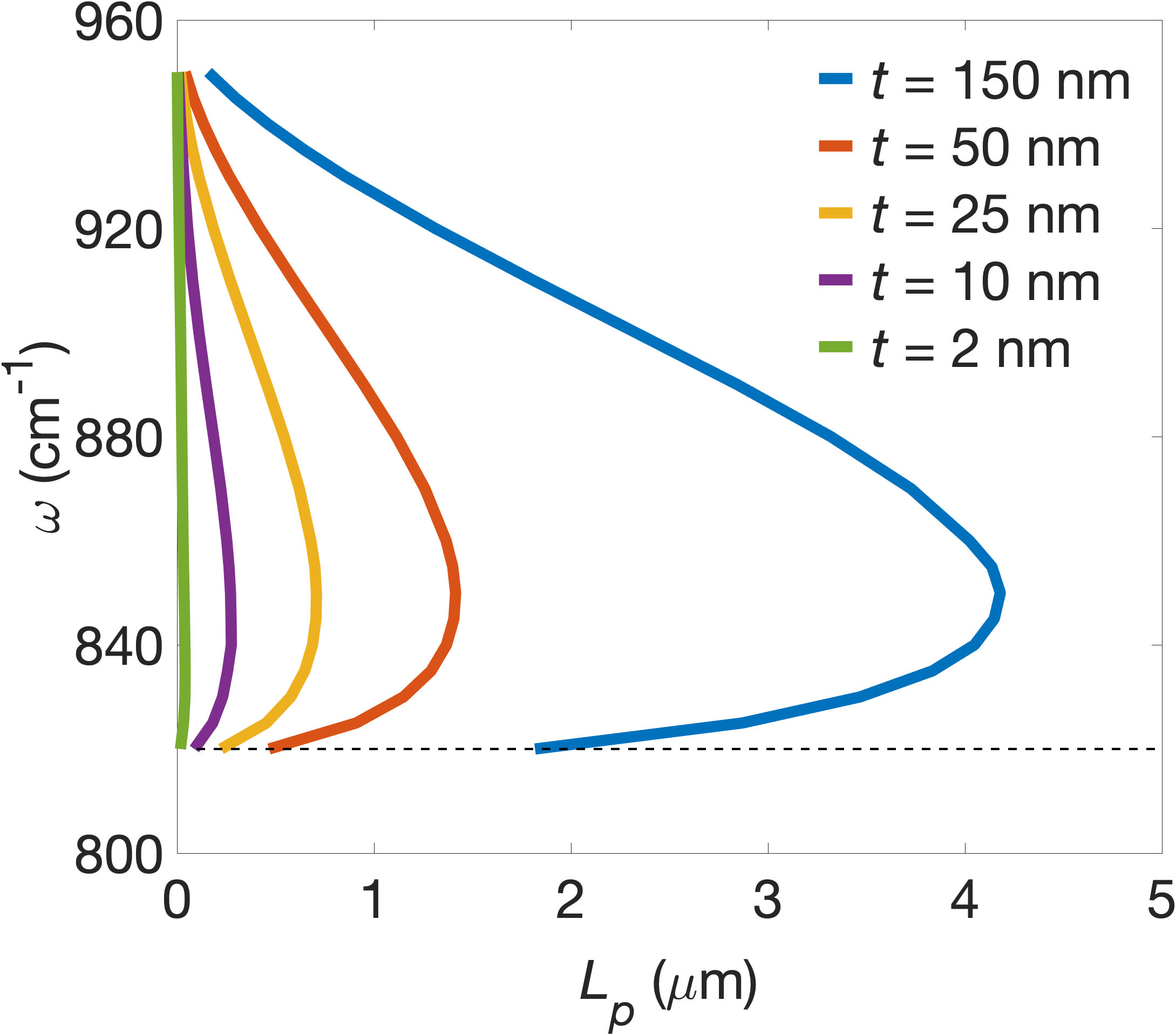


(A) (B)


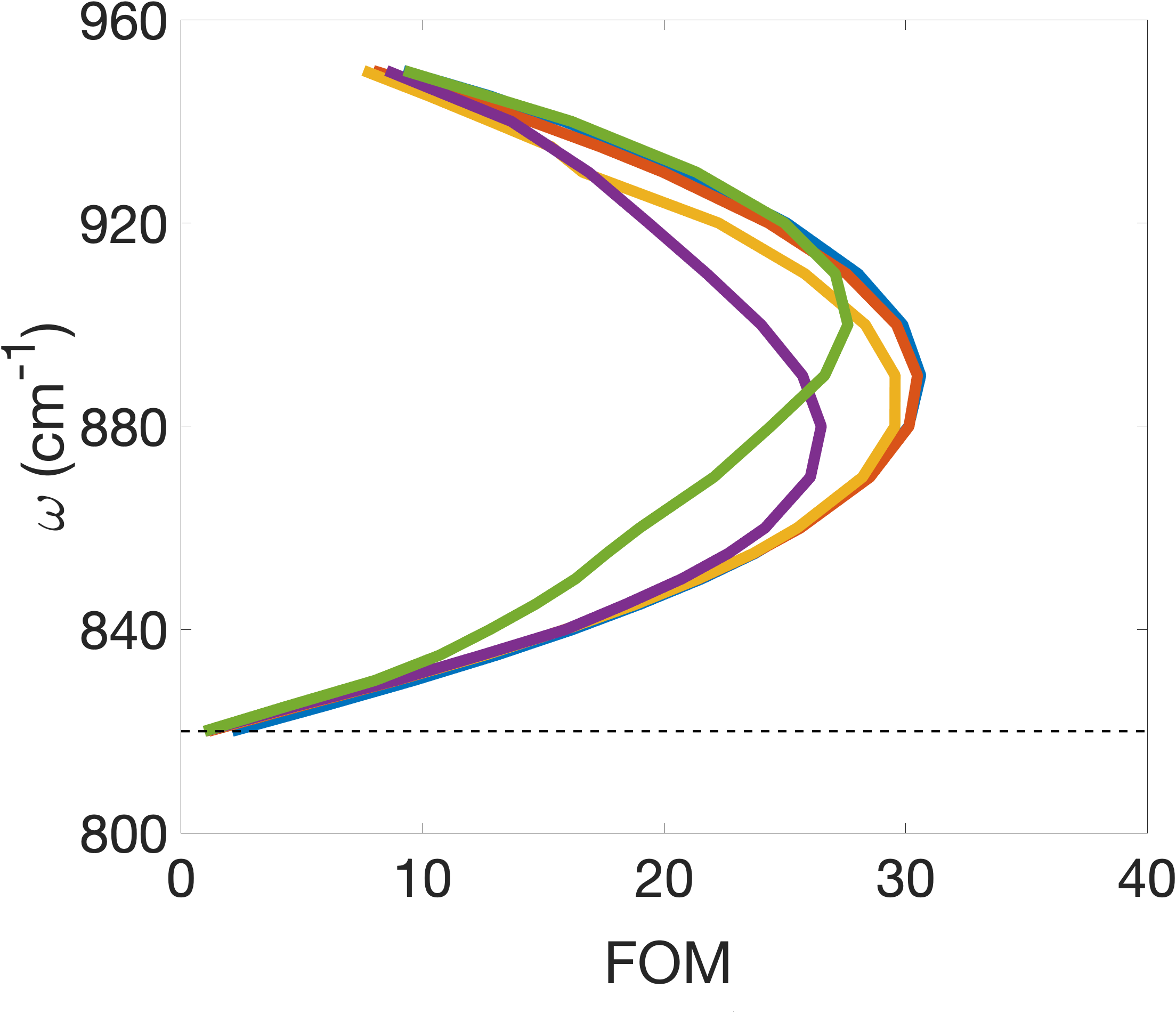

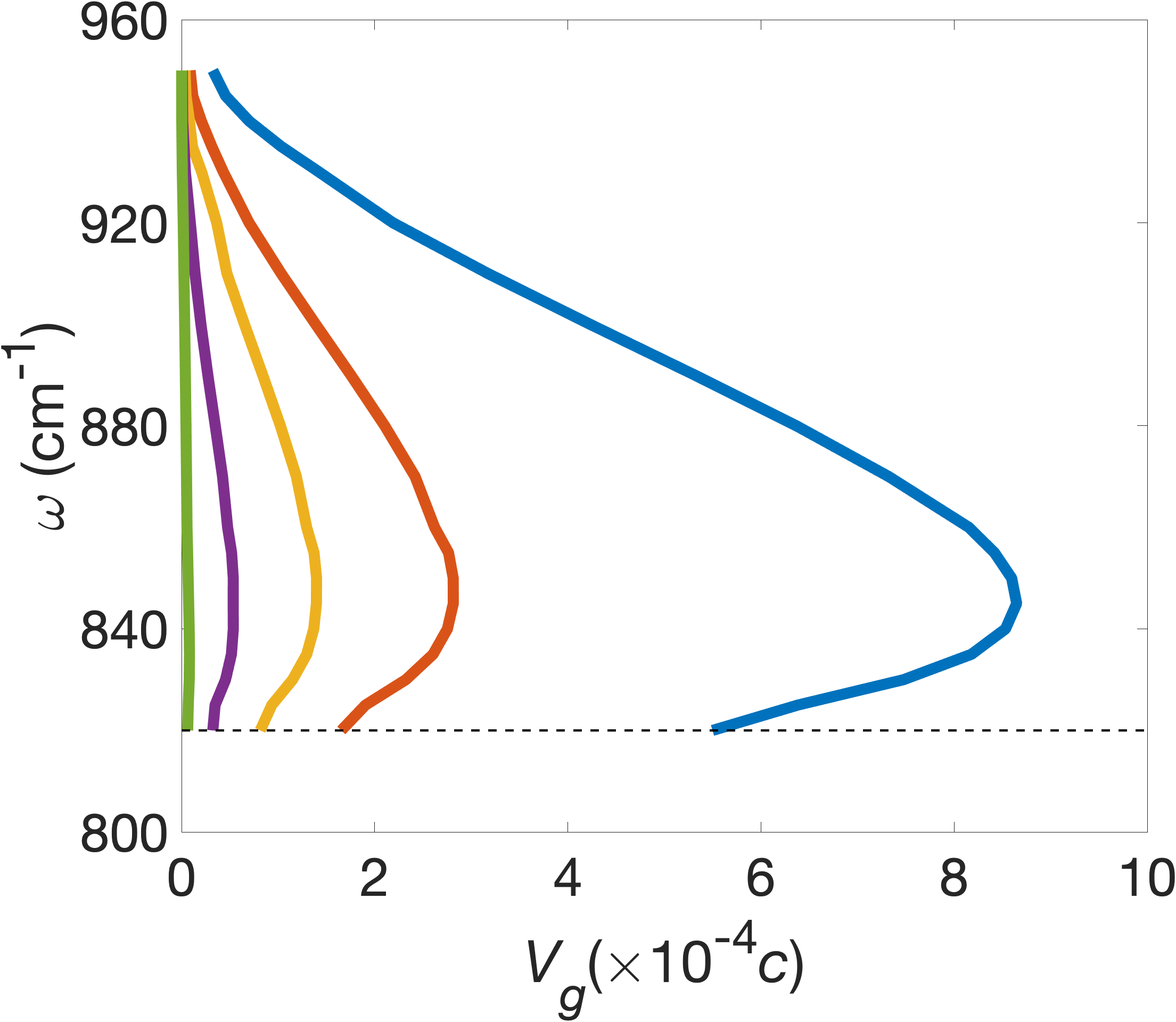


(C) (D)

**Fig. S2:** Wavenumber *ω* for various thicknesses of the *α*-MoO_3_ slab without a graphene cover against (A) the real part of the wavenumber along the *x*-direction Re(*k_x_*), (B) the propagation length *L_p_*, (C) the benefit-to-cost ratio FOM, and (D) the group velocity *v_g_*.

(A) (B) (C)

**Fig. S3:** *E_z_* field profiles of the *α*-MoO_3_ slab without graphene at *t* = (A) 150 nm, (B) 50 nm, and (C) 10 nm. (C) also shows a magnified view in the upper right corner for clearer observation.

1. Re(*E_z_*) field distributions and IFCs
   1. Of an *α*-MoO_3_ slab rotated by the angle *θ*

**Figure S4A** depicts the location of the dipole on the *x*–*y* plane, and the scale bar in all figures represents 1 µm.


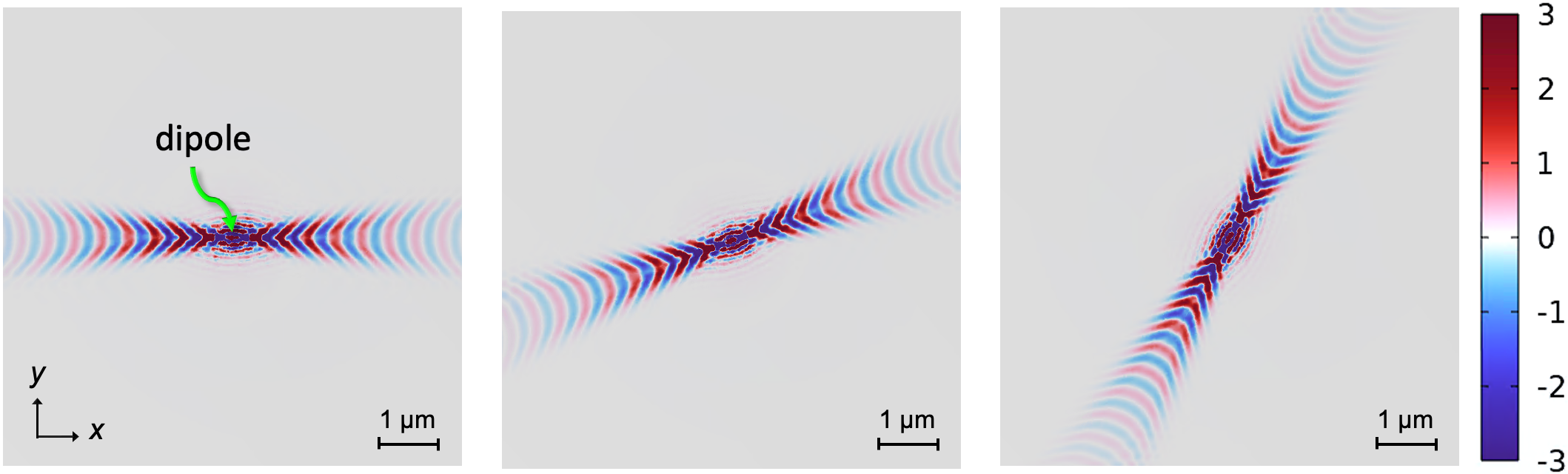


(A) (B) (C)


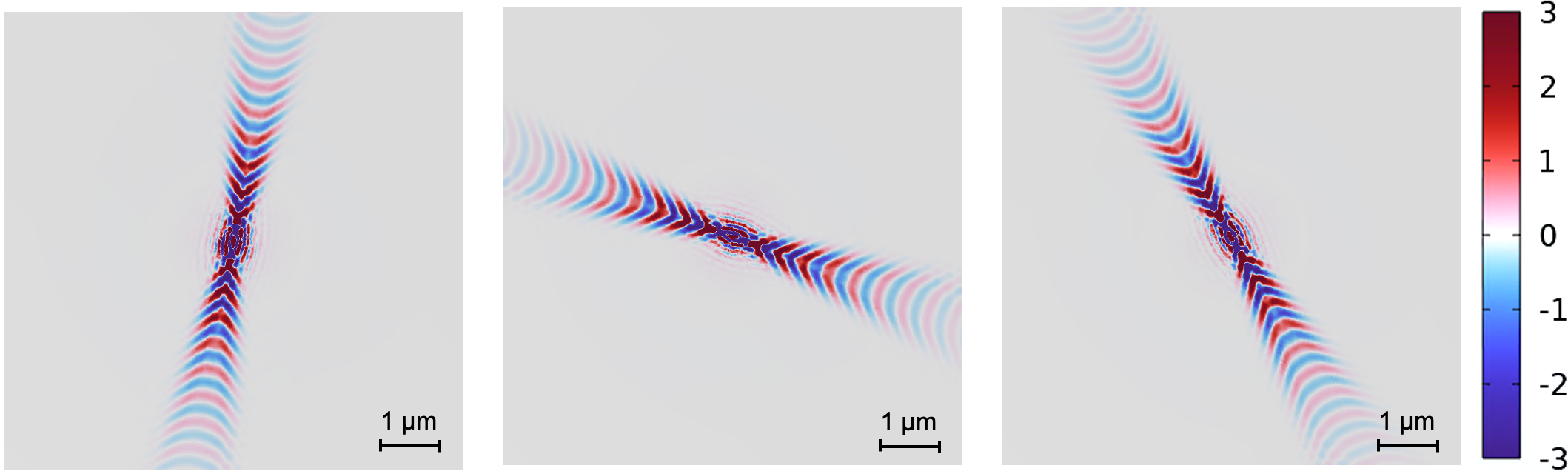


(D) (E) (F)


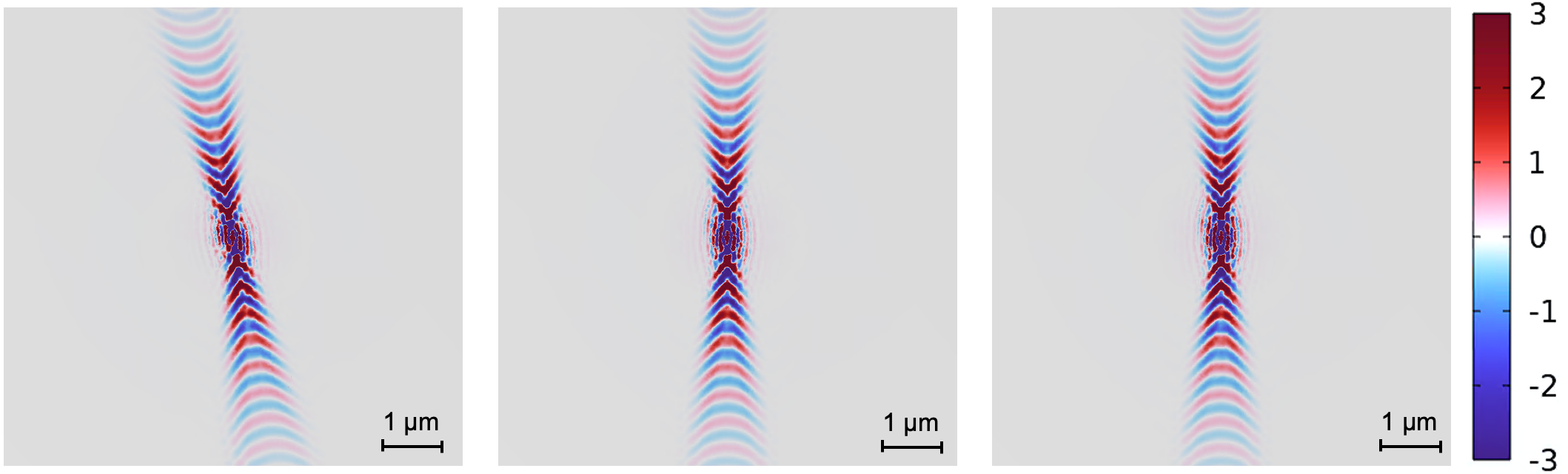


(G) (H) (I)

**Fig. S4:** Field distributions of Re(*E_z_*) at various angles *θ*: (A) 0°, (B) 20°, (C) 60°, (D) 80°, (E) −20°, (F) −60°, (G) −80°, (H) 90°, and (I) −90°. Simulations were conducted by using a fixed thickness *t* = 150 nm for the *α*-MoO_3_ slab, frequency of *ω* = 910 cm^−1^, and Fermi energy of *E_f_* = 0.15 eV. The scale bar represents 1 µm.


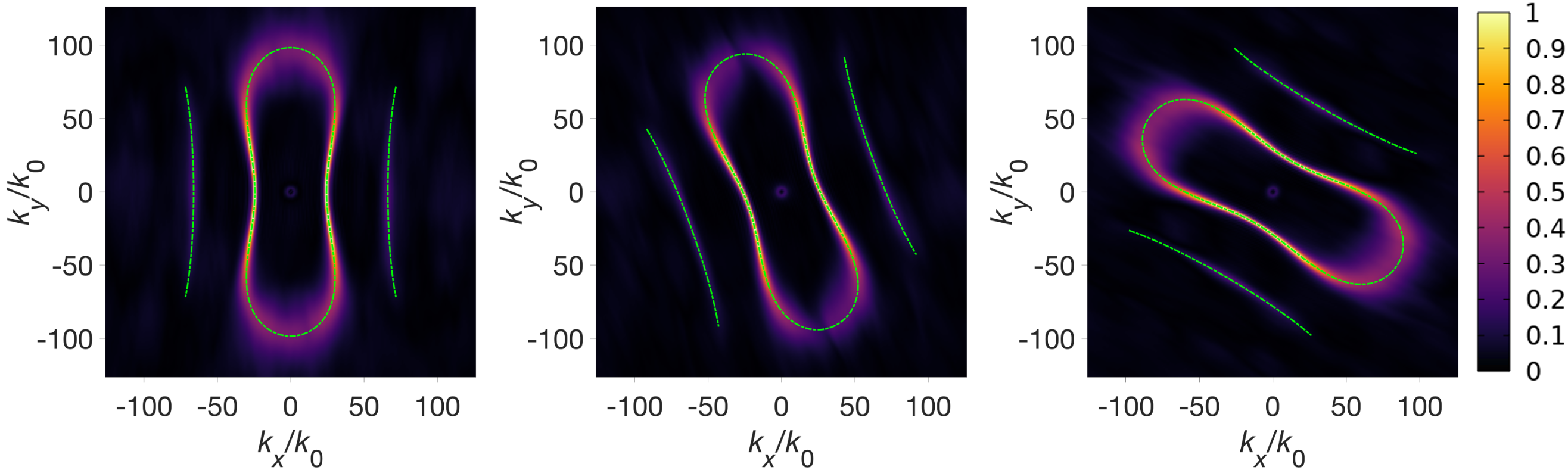


(A) (B) (C)


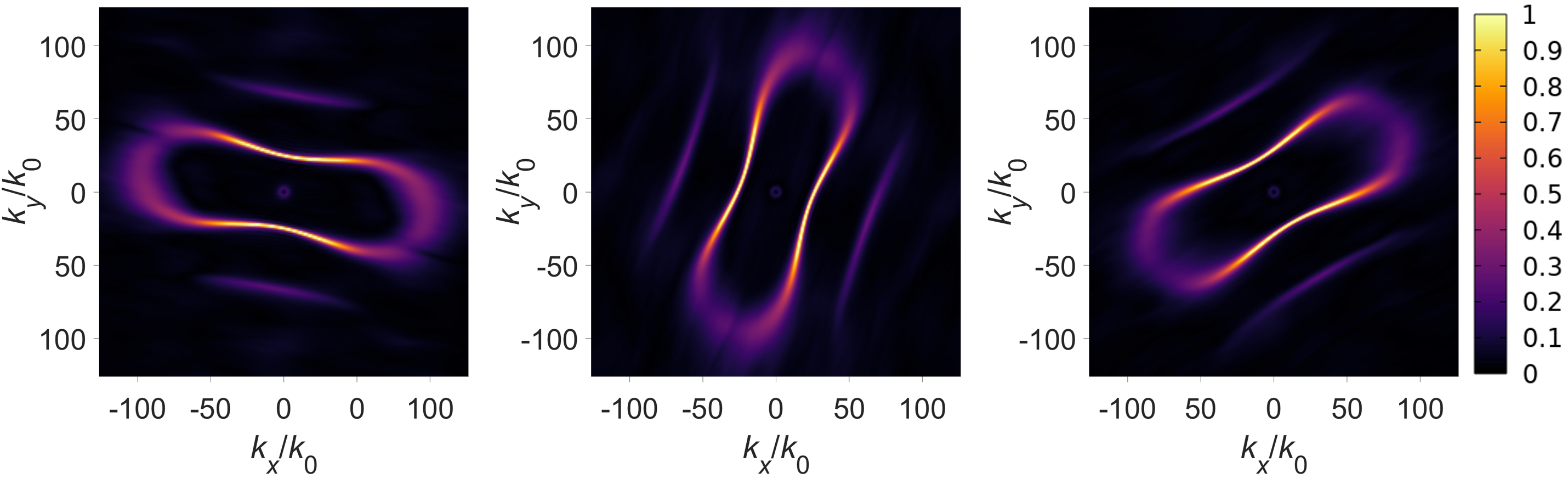


(D) (E) (F)


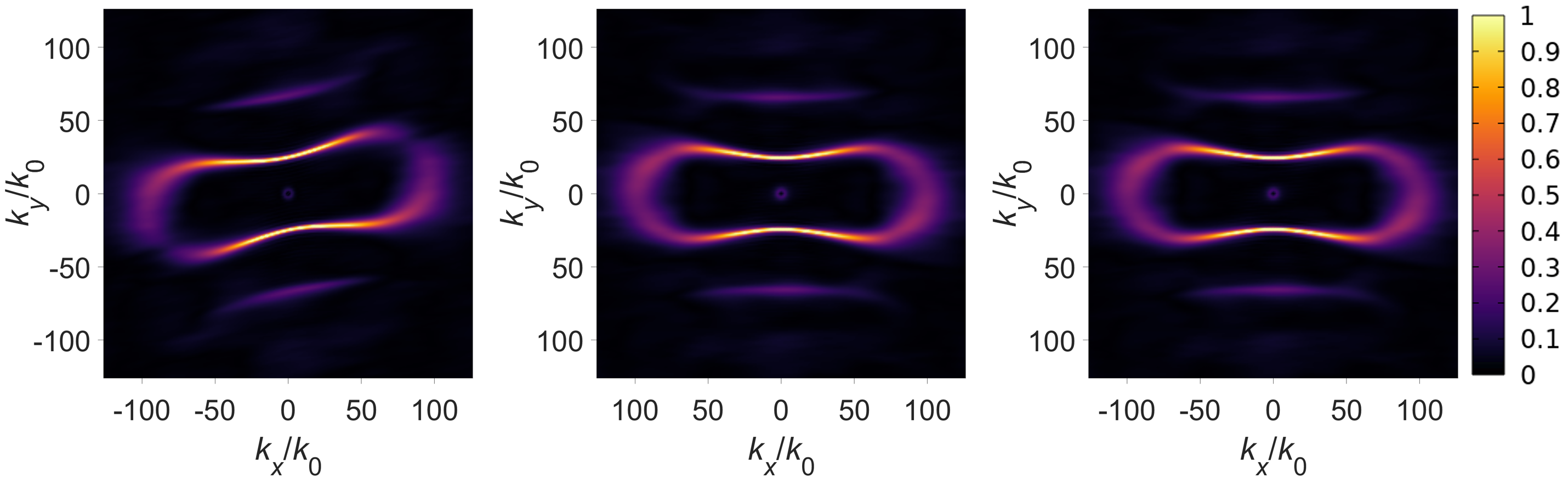


(G) (H) (I)

**Fig. S5:** Isofrequency contours (IFCs) obtained by the Fourier transform of Re{*E_z_*} under the conditions of *t* = 150 nm, *ω* = 910 cm^−1^, and *E_f_* = 0.15 eV at the angles *θ* of (A) 0°, (B) 20°, (C) 60°, (D) 80°, (E) −20°, (F) −60°, (G) −80°, (H) 90°, and (I) −90°, where the green dashed lines indicate the analytical calculated IFCs.

- 1. Of a bare *α*-MoO_3_ slab and graphene/*α*-MoO_3_ slab with different values of *E_f_* on an Au substrate at *θ* = 20°


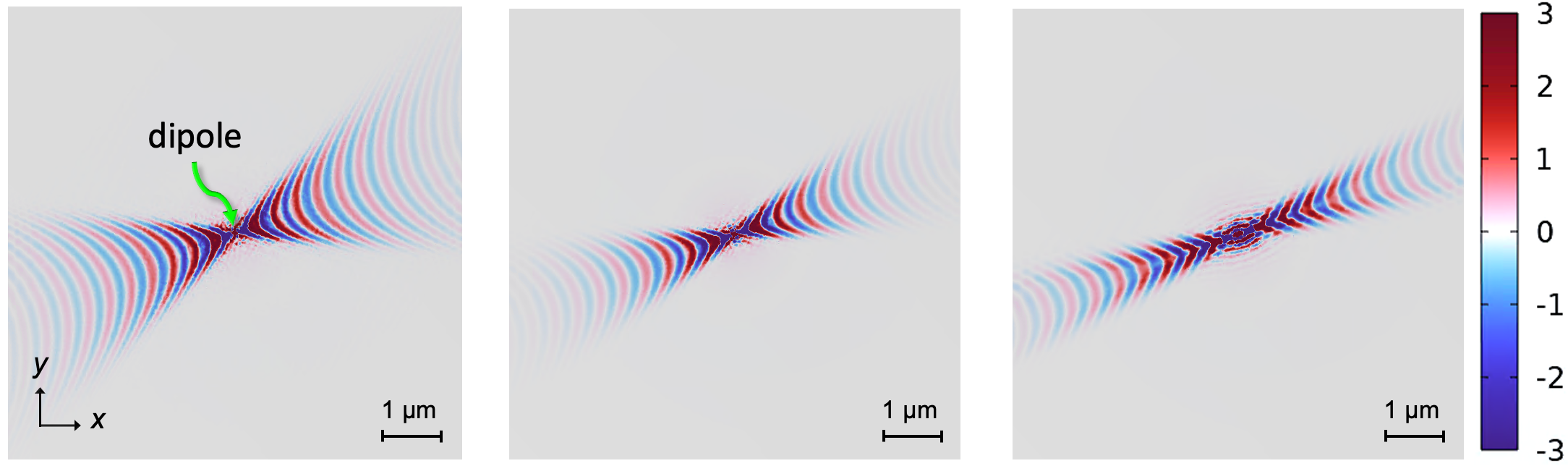


(A) (B) (C)


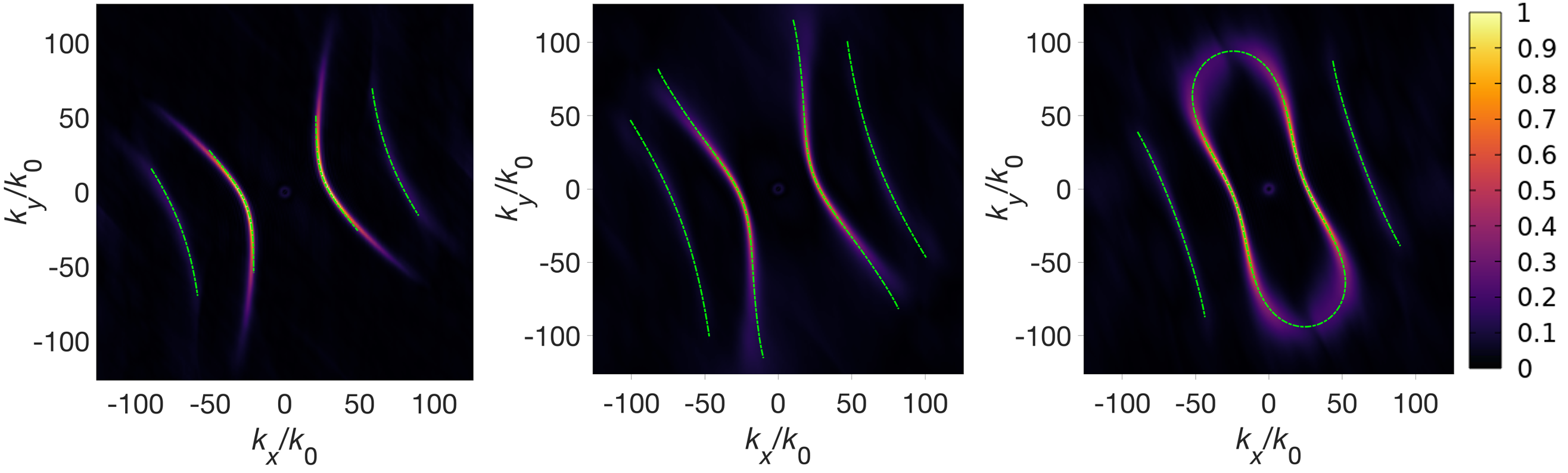


(D) (E) (F)


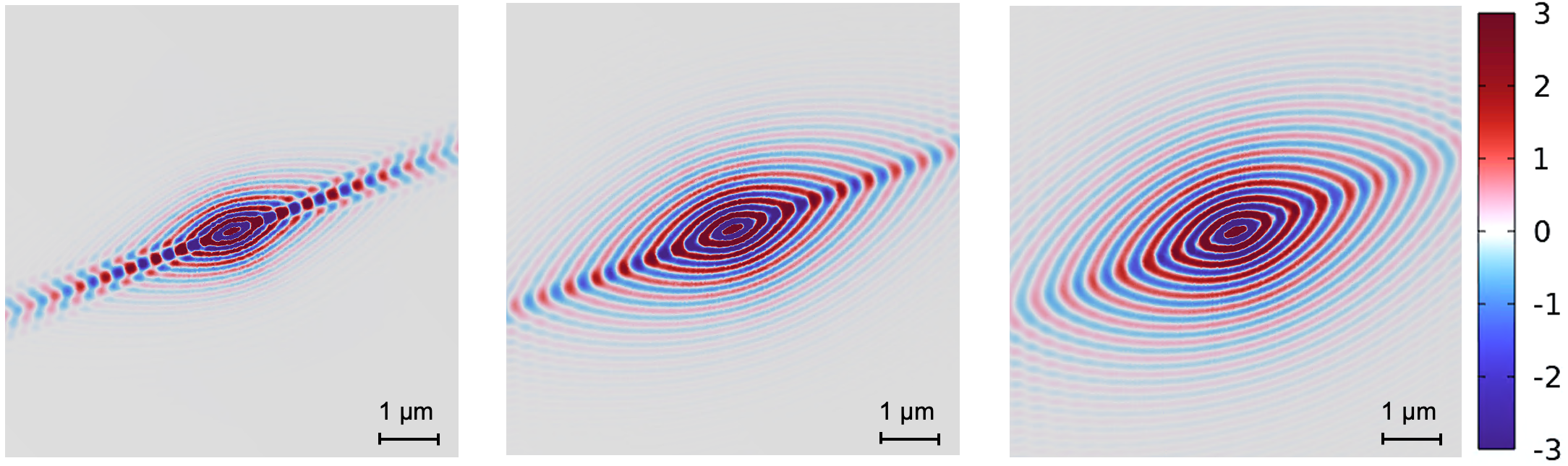


(G) (H) (I)


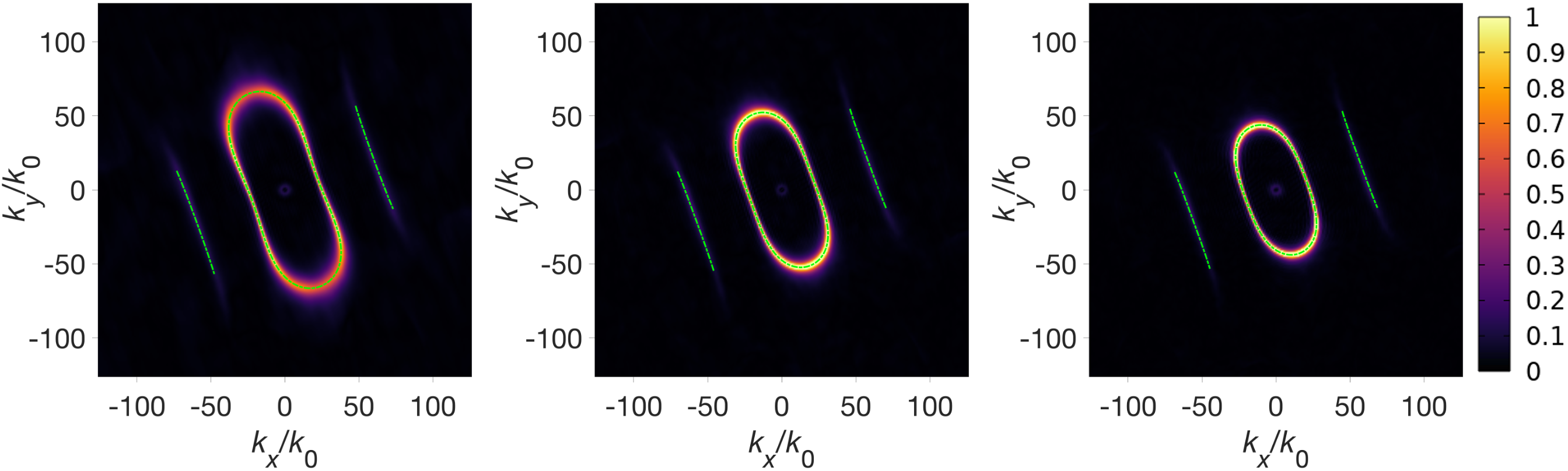


(J) (K) (L)

**Fig. S6:** Re(*E_z_*) field distributions for (A) no graphene and *E_f_* = (B) 0.1, (C) 0.15, (G) 0.2, (H) 0.25, and (I) 0.3 eV. IFCs for (D) no graphene and *E_f_* = (E) 0.1, (F) 0.15, (J) 0.2, (K) 0.25, and (L) 0.3 eV, where the green dashed lines indicate the analytical calculated IFCs.

1. Two differently oriented *α*-MoO_3_ slabs covered with graphene layer
   1. Re(*E_z_*) field distributions and IFCs with different values of *t* at *E_f_* = 0.15 eV, *θ*_1_ = 20°, and *θ*_2_ = −30°

(A) (B) (C)


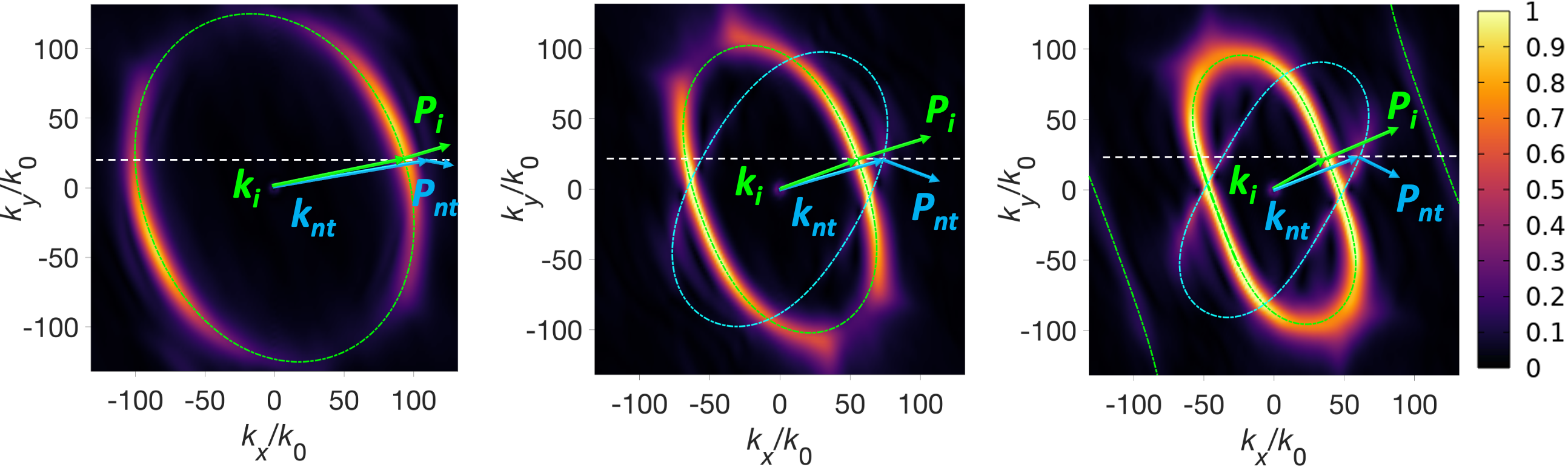


(D) (E) (F)

(G) (H) (I)


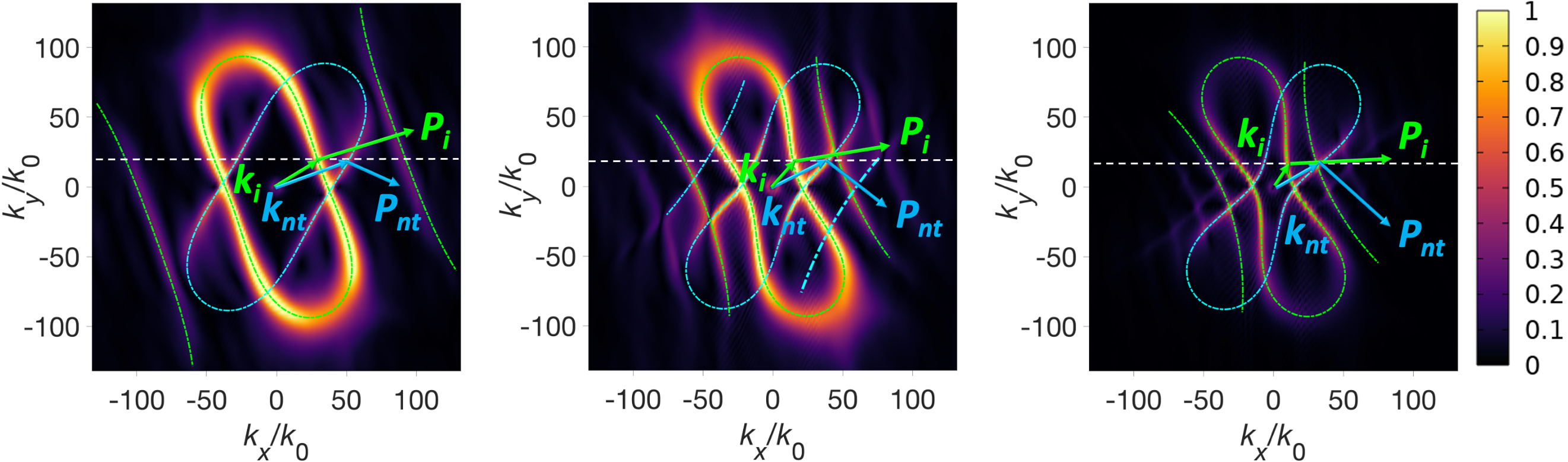


(J) (K) (L)

**Fig. S7:** Re(*E_z_*) field distributions under the conditions of *ω* = 910 cm^−1^, *E_f_* = 0.15 eV, *θ*_1_ = 20°, and *θ*_2_ = −30° at *t* of (A) 25, (B) 50, (C) 75, (G) 100, (H) 200, and (I) 300 nm and (D)–(L) the corresponding IFCs, where the green and cyan dashed lines indicate the analytical calculated IFCs of the left and right *α*-MoO_3_ slabs, respectively.

- 1. Re(*E_z_*) field distributions and IFCs with different values of *ω* at *t* = 150 nm, *E_f_* = 0.15 eV, *θ*_1_ = 20°, and *θ*_2_ = −30°

(A) (B) (C)

(D) (E) (F)


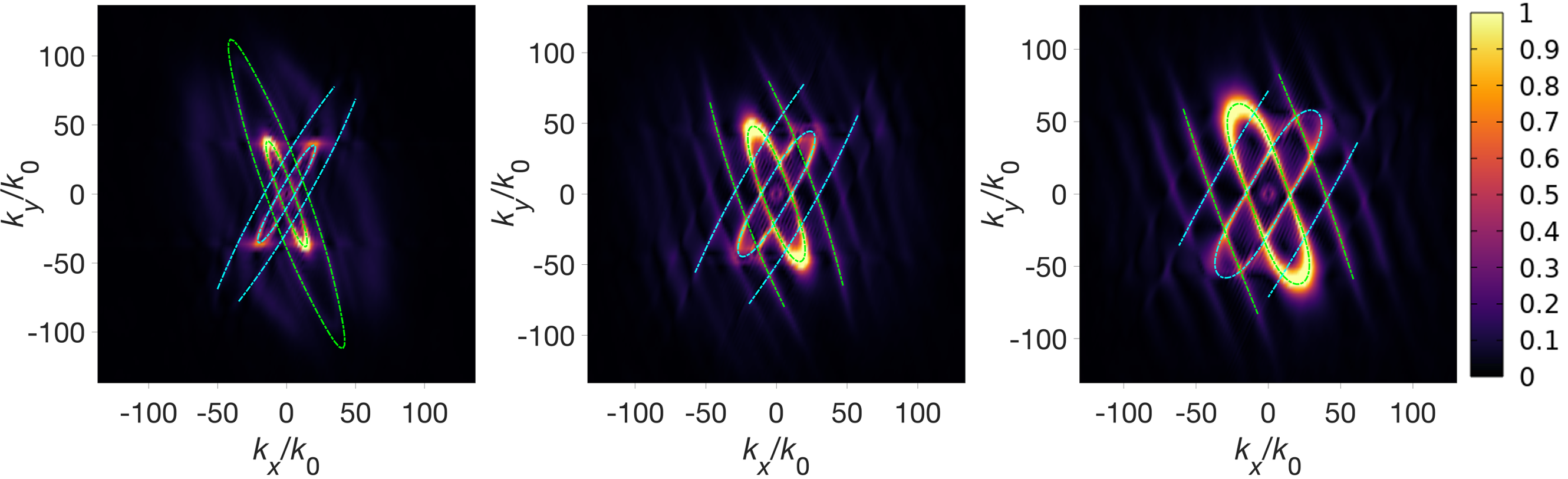


(G) (H) (I)


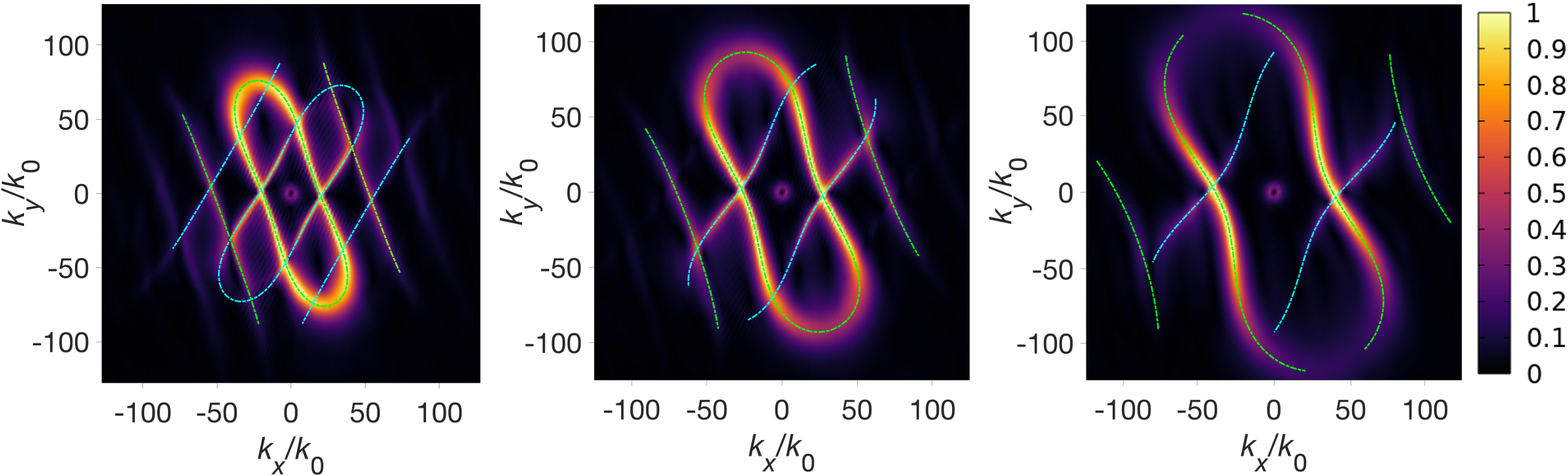


(J) (K) (L)

**Fig. S8:** Re(*E_z_*) field distributions at *t* = 150 nm, *E_f_* = 0.15 eV, *θ*_1_ = 20°, and *θ*_2_ = −30° for *ω* = (A) 830, (B) 850, (C) 870, (D) 890, (E) 910, and (F) 930 cm^−1^. Corresponding IFCs are shown in (G)–(L), respectively, where the green and cyan dashed lines indicate the analytical calculated IFCs of the left and right *α*-MoO_3_ slabs, respectively.

1. ***Corresponding author:** **Chia-Chien Huang**, Department of Physics and Graduate Institute of Nanoscience, National Chung Hsing University, Taichung, Taiwan; [cch@phys.nchu.edu.tw](mailto:cch@phys.nchu.edu.tw); **Ruey-Tarng Liu:** Department of Physics, National Chung Hsing University, Taichung, Taiwan. [↑](#footnote-ref-1)
